# Concepts of dementia prevention in the health promotion among older adults: A narrative review

**DOI:** 10.1097/MD.0000000000032172

**Published:** 2022-12-16

**Authors:** Fu-Ju Tsai, Sheng-Wei Shen

**Affiliations:** a Department of Nursing, Fooyin University, Taiwan R.O.C.; b Department of Neurology, Pingtung Hospital, Ministry of Health and Welfare, Taiwan, R.O.C.

**Keywords:** dementia prevention, health promotion, older adults, preventive strategies

## Abstract

The number of older adults with dementia is predicted to markedly increase in the coming decades. A person suffers from dementia every 3 seconds globally, and one out of every 7 people suffers from dementia in Taiwan. The purpose of this narrative review is to integrate existing concepts of dementia prevention into health promotion and improve older adults’ quality of life. This narrative review was performed using the PubMed database by searching for basic research and systematic reviews on dementia prevention and health promotion among older adults. We established a framework for dementia prevention and health promotion with regard to the physical, mental, spiritual, and social health aspects. We identified the following strategies related to older adults towards dementia prevention and health promotion in follows: Physical health promotion: cognitive activities, physical activities, body mass index, balanced diet, rainbow diet, Mediterranean diet, dietary approaches to stop hypertension diet, mind diet, no smoking and drinking, avoiding the “three highs” (i.e., hyperglycemia, hyperlipidemia, and hypertension), and head trauma; Mental health promotion: Positive thinking, Brief Symptom Rating Scale (BSRS-5), depression scale, and ascertained dementia 8 questionnaire (AD8) screening; Spiritual health promotion: religious beliefs, spiritual music, meditative activities, mindfulness, yoga, Qi-gong, Tai-chi, and Baduanjin; and Social health promotion: A supportive family system, socialization, social support, social networks, social interaction, and social participation. The conclusion of this narrative review was to integrate the concepts of dementia prevention and health promotion among older adults.

## 1. Introduction

Globally, one person suffers from dementia every 3 seconds. In Taiwan, 1 out of every 7 people suffer from dementia. According to the Taiwan Ministry of Health and Welfare in 2018, the prevalence rate of dementia in adults over 65 years of age was 8%. The current prevalence rate of dementia is 3.40% among those 65 to 69 years old, 3.46% among those 70 to 74 years old, 7.19% among those 75 to 79 years old, 13.03% among those 80 to 84 years old, 21.92% among those 85 to 89 years old, and 36.88% among those 90 years and older.^[1]^

Identified risk factors for dementia include hypertension, hyperglycemia and obesity, smoking, depression, physical inactivity, and social isolation.^[2,3]^ Dementia prevention and health promotion efforts are directed towards older adults to provide them with a higher quality of life. Increasing emphasis has been placed on numerous strategies for dementia prevention, including physical, mental, spiritual, and social health promotion. Indeed, dementia prevention and health promotion will be increasingly crucial among older adults globally in the foreseeable future.^[4]^

Very early screening for dementia may be performed using a scale comprising 8 questions.^[5]^ The 8 items were as follows: difficulty in judgment; decreased interest in activities and hobbies; repeating the same questions, stories, and/or statements; difficulty in learning how to use instruments, equipment, and devices; forgetting the correct month and year; difficulty handling complex objects; difficulty remembering appointments; and persistent thinking and memory problems. On this scale, if 2 or more of the above items are or become present, that person is advised to seek medical treatment as soon as possible.^[5]^

Regarding health beliefs and health promotion, many strategies exist to prevent dementia among older adults.^[6]^ These include physical, mental, spiritual, and social health promotion aspects^[4]^ such as cognitive activities, body mass index (BMI) control, Mediterranean diet, vitamin D supplementation,^[7]^ no smoking, no drinking, avoiding the 3 highs (i.e., hyperglycemia, hyperlipidemia, and hypertension),^[8]^ avoidance of depression, engagement in physical activity,^[9]^ maintenance of a generally happy mood, and engagement in social participation.^[3]^ These health promotion strategies aim to enhance dementia prevention among older adults in their daily lives.

The incidence of dementia has continued to increase and greatly affects older Taiwanese adults. Owing to the current prevalence of dementia, it is hoped that Taiwanese older adults will receive efficacious health beliefs, health promotion strategies, and dementia prevention in the future.^[10]^ In this narrative review, we organized many numerous strategies for dementia prevention, health beliefs, and health promotion with regard to physical, mental, spiritual, and social health promotion among older adults.^[11,12]^

### 1.1. Purpose

The purpose of this narrative review is to integrate the concepts of dementia prevention in physical, mental, spiritual, and social health aspects among older adults to promote their quality of life for delaying aging process.

## 2. Methods

This narrative review was performed using the PubMed database by searching the basic literature and systematic reviews on dementia prevention and health promotion among older adults. Studies from other databases were excluded. We established a framework for dementia prevention and health promotion with regard to the physical, mental, spiritual, and social health aspects.

### 2.1. Searching strategy

We used 2 keywords or combinative keywords and searched in the PubMed database as follows: “physical promotion and dementia,” “mental promotion and dementia,” “spiritual promotion and dementia,” “social aspects of health and dementia promotion,” “cognitive activities and dementia prevention,” “physical activities and dementia prevention,” “BMI and dementia prevention,” “balanced diet and dementia prevention,” “Rainbow diet and dementia prevention,” “Mediterranean diet and dementia prevention,” “DASH diet and dementia prevention,” “Mind diet and dementia prevention,” “no smoke or smoking and dementia prevention,” “no drink or drinking and dementia prevention,” “hyperglycemia and dementia prevention,” “hyperlipidemia and dementia prevention,” “hypertension and dementia prevention,” “head trauma or injury and dementia prevention,” “positive thinking and dementia prevention,” “BSRS-5 and dementia prevention,” “depression scale and dementia prevention,” “AD8 screening and dementia prevention,” “religious beliefs and dementia prevention,” “spiritual music and dementia prevention,” “meditative activities and dementia prevention,” “mindfulness and dementia prevention,” “yoga and dementia prevention,” “Qi-gong and dementia prevention,” “Tai-chi and dementia prevention,” “Baduanjin and dementia prevention,” “supportive family system and dementia prevention,” “socialization and dementia prevention,” “social support and dementia prevention,” “social network and dementia prevention,” “social interaction and dementia prevention,” and “social participation and dementia prevention.”

### 2.2. Study selection

We searched for many articles and selected relevant articles for reading based on the title, abstract, keywords, and text. After reading many articles that were associated with dementia prevention, health promotion, and older adults, we finally used 88 articles from 2008 to 2021 in this study. This narrative review did not require ethical approval because no human data were used in the study.

### 2.3. Concepts of dementia prevention and health promotion

**Figure 1.**
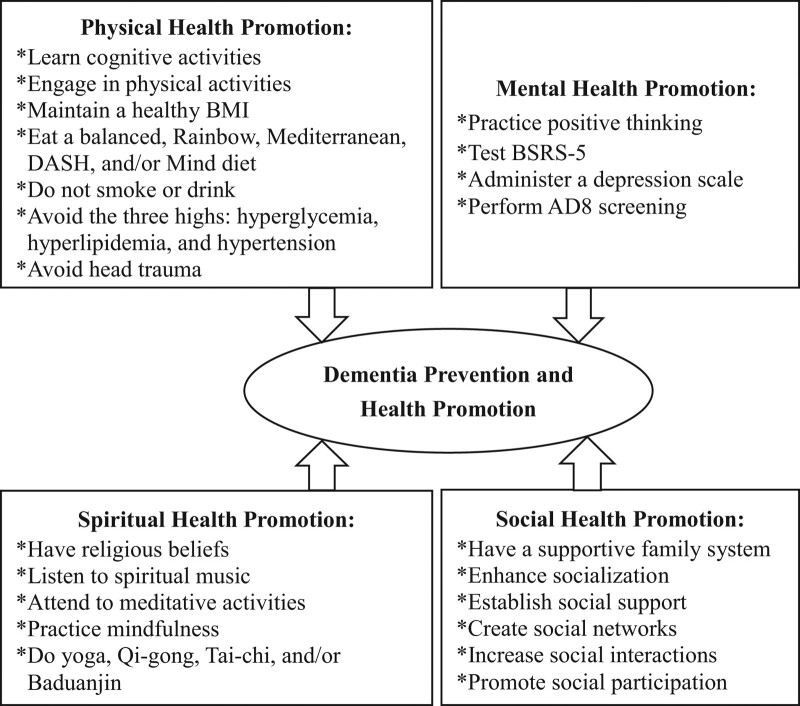
Framework of dementia prevention and health promotion. It indicated dementia prevention and health promotion in physical, mental, spiritual, and social health promotion.

We used this framework to organize the concepts of dementia prevention and health promotion (Fig. 1), comprising physical, mental, spiritual, and social health aspects to achieve and maintain healthy lifestyles and life experiences.^[13,14]^

Dementia prevention and physical health promotion included several health promotion strategies that were related to learning cognitive activities, performing physical activities, maintaining a healthy BMI, eating a balanced, rainbow, Mediterranean, dietary approaches to stop hypertension (DASH), and/or Mind diet,^[15]^ not smoking tobacco or drinking alcohol, avoiding the 3 highs (i.e., hyperglycemia, hyperlipidemia, and hypertension), and avoiding head trauma.^[1]^

Dementia prevention and mental health promotion comprise various methods in health promotion, such as practicing positive thinking, testing BSRS-5,^[16,17]^ performing assessments using a depression scale, and utilizing AD8 screening.^[1]^

Dementia prevention and spiritual health promotion include religious beliefs, listening to spiritual music, engaging in meditative activities, maintaining mindfulness, and performing yoga, Qi-gong, Tai-chi, and Baduanjin.^[1]^

Dementia prevention and social health promotion comprise having a supportive family system, enhancing socialization, establishing social support, creating a social network, increasing social interaction, and promoting social participation.^[1]^

## 3. Results

### 3.1. Physical health promotion with dementia prevention

#### 3.1.1. Learn cognitive activities.

Cognitive and creative activities may reduce the risk of dementia by 50% and enhance brain function to facilitate remaining curious and learning new knowledge.^[1]^ One study showed that improved cognitive function among older adults can be achieved through preventive interventions.^[18]^ Indeed, continuing to practice and learn new cognitive activities can significantly slow cognitive decline and dementia among older adults. Strategies that involve cognitive activities contribute to preventing the aging process associated with dementia in daily life.^[19]^ In Taiwan, health promotion strategies aimed at increasing cognitive activities include doing puzzles, playing chess, memory flops, playing cognitive games, reading books, reading newspapers, reading magazines, etc.^[1]^

#### 3.1.2. Engage in physical activities.

Conducting regular physical activities more than twice per week for at least 150 minutes per week has a protective effect that decreases the risk of dementia by 60%. Such physical activities include walking, climbing, swimming, cycling, going to the gym, aerobics, and calisthenics.^[1]^ A study reported that physical activity may decrease dementia symptoms in daily life.^[20]^ In fact, physical activity can be used in many strategies for dementia prevention and health promotion among older adults. A study provides a narrative review of the current evidence on dementia intervention with physical activity to promote cognitive health among older adults.^[21]^ Healthy lifestyles in older adults, such as leisure activities, physical activities, and physical exercise, constitute protective interventions against dementia with cognitive decline.^[22]^ Therefore, physical activity and exercise interventions may be effective in preventing neurological damage and dementia in older adults.^[23]^

#### 3.1.3. Maintain a healthy BMI.

Older adults with BMI ≧ 30 are at a 3 times greater risk of dementia. Being overweight in older adults with a BMI between 25 and 30 increases the risk of dementia 2-fold. In addition, older adults with BMI of less than 18 are also associated with an increased risk of dementia. Therefore, older adults should maintain a healthy body weight with a BMI between 18.5 and 24 to decrease the risk of dementia.^[1]^ BMI is also associated with dementia in older adults with cardiovascular risk factors.^[24,25]^ Among older adults, higher BMI and obesity are proven risk factors for dementia and cognitive impairment.^[26]^ Controlling BMI and body weight in older adults is critical for maintaining cognitive and physical functions for dementia prevention.^[27]^ Dementia prevention is positively correlated with BMI and cognitive function in older adults.^[28]^ Recent literature indicates that BMI and dementia prevention are associated with older adults’ body weight and BMI measurements in daily life.^[29]^ Obesity and BMI are also risk factors for stroke and are associated with dementia in older adults.^[30]^ Since older adults with an elevated BMI are at an increased risk of dementia, they should control their body weight and BMI to prevent dementia.^[31]^ Therefore, a BMI outside the normal range is correlated with dementia and cognitive impairment in older men and women.^[32–34]^

#### 3.1.4. Eat a balanced, rainbow, mediterranean, DASH, and/or mind diet.

Concerning the prevention of dementia, Mediterranean and Mind diets have been shown to reduce the prevalence of dementia among older adults.^[35]^ The dietary components of the Mediterranean diet have been reviewed in terms of the aging process, cognitive decline, and dementia prevention.^[36]^ It has been demonstrated that the Mediterranean and DASH diets slow cognitive decline and contribute to dementia prevention among older adults.^[37]^ The current study provides an overview of the evidence associated with the Mediterranean, DASH, and Mind diet interventions for dementia prevention. A higher intake of foods that adhere to the Mediterranean, DASH, and/or Mind diets is associated with good cognitive function in older adults, and a higher intake of foods that adhere to the DASH or Mind diets is also related to dementia prevention among older adults.^[38]^ In addition, an integral component of the rainbow diet is fruits such as grapes and vegetables such as spinach, which are positively correlated with dementia prevention and health promotion in older adults.^[39]^ A literature review shows that an appropriate carnitine-rich diet, such as the Mediterranean, DASH, and Mind diets, contributes to dementia prevention in older adults. Furthermore, a balanced diet containing essential amino acids and carnitine synthesis associated with fruits and vegetables supports dementia prevention. Fruits and vegetables are also essential components of the Mediterranean diet and contribute to the prevention of dementia.^[40,41]^ The Mediterranean diet is characterized by a high consumption of fruits and vegetables, cereals, nuts, olive oil, seeds, fish, poultry, dairy products, and little alcohol. In the mind diet, food that is rich in carnitine or carnitine supplementation may improve older adults’ cognitive function in everyday life.^[42]^ Overall, it has been proven that older adults who consume balanced, rainbow, Mediterranean, DASH, and/or mind diets achieve beneficial results regarding physical health promotion and dementia prevention.

#### 3.1.5. Do not smoke and drink.

Among older adults, smoking tobacco and drinking alcohol are risk factors for dementia and are associated with twice the risk of cognitive decline.^[1]^ Older adults who continue to smoke also tend to experience a faster rate of cognitive decline each year.^[1]^ A previous study explored the association between the risk factors of smoking and dementia.^[43]^ Older adults at the highest risk of dementia were found to smoke and drink alcohol the most. As overall smoking and alcohol use are related to an increased risk of dementia, it is necessary to reduce smoking and drinking to support dementia prevention.^[44,45]^ No smoking or drinking is a strong predictor of dementia prevention among older adults.^[46]^

#### 3.1.6. Avoid the 3 highs: hyperglycemia, hyperlipidemia, and hypertension.

Diabetes, cardiovascular disease, stroke, and hypertension increase the risk of dementia in older adults.^[1]^ Several studies have indicated that diabetes may cause memory or cognitive decline. Systolic blood pressure > 160 mm Hg and untreated hypertension are associated with a 5-fold increase in the risk for dementia. Controlling high BP levels has also been shown to reduce dementia risk.^[1]^ Consequently, older adults with diabetes, hyperlipidemia, and hypertension should receive treatment as soon as possible to maintain blood sugar, blood lipids, and blood pressure within the normal range for dementia prevention and physical health promotion.^[1]^ No history of diabetes, history of hypertension, and a healthy lifestyle are strong predictors of dementia prevention and physical health promotion among older adults.^[46]^ It is essential that older adults at high risk of dementia engage in preventive strategies such as regular exercise, normal sleep, and no smoking and drinking associated with healthy lifestyles to achieve and maintain normal blood sugar, blood lipids, and blood pressure in their daily lives.^[47]^ Indeed, it is clear related to healthy lifestyles that older adults should avoid the 3 highs of hyperglycemia, hyperlipidemia, and hypertension to prevent dementia.^[46,47]^

#### 3.1.7. Avoid head trauma.

Head trauma is a major risk factor of dementia. Older adults with severe brain damage are 4 times more likely to develop dementia.^[1]^ Avoiding the possibility of head trauma contributes to the prevention of dementia development.^[48]^ Certain environmental factors that may increase the probability of head trauma must be controlled to prevent dementia.^[49]^ The adoption of healthy lifestyles that contain strategies for avoiding head trauma among older adults in their everyday lives is imperative.^[49]^

### 3.2. Mental health promotion with dementia prevention

#### 3.2.1. Practice positive thinking.

A study surveyed the effects of cognitive function on dementia prevention among older adults.^[50]^ Positive thinking is an effective strategy for dementia prevention in older adults. On the other hand, negative thinking is related to mood problems such as depression. In addition, a lack of social relationships can indicate that older adults have negative beliefs, lower life satisfaction, lower self-esteem, and lower social participation.^[50]^ In aggregate, extant literature demonstrates that older adults need to have positive thinking in their daily lives to achieve dementia prevention.^[50]^

#### 3.2.2. Test BSRS-5.

The BSRS-5 is a simple 5-item questionnaire that assesses emotional distress for mental health promotion.^[16,17]^ The BSRS-5, which is also commonly known as a “Mood thermometer,” is used for mental health symptom screening among older adults.^[16,17]^ This assists in understanding older adults’ moods to prevent emotional distress. The BSRS-5 is a self-filling scale and supports older adults with their self-health management in daily life.^[16,17]^ Emotional distress may produce mental symptoms of depression, which have been shown to be a risk factor for dementia. Therefore, older adults’ emotional states should be examined to prevent dementia.^[16,17]^

#### 3.2.3. Administer a depression scale.

Older adults with depression are at markedly high risk for dementia. Many investigations have shown that older adults who are depressed are at twice the risk of developing dementia.^[1]^ Consequently, older adults with depression should receive treatment to prevent dementia. A balanced diet and treatment of mental health problems are suggested, and fruits and vegetables have a high content of nutrients to promote good mental health. A balanced diet is related to avoiding depressive disorders, improving mental health, and preventing dementia. Indeed, greater consumption of fruits and vegetables in daily life is reported to have a significantly positive influence on mental health, and thus, a decreased incidence of dementia.^[51]^ One study indicated that older adults with late-life depression were associated with a greater incidence of dementia. Therefore, implementation of a depression scale is a simple and effective strategy and intervention to prevent dementia development.^[52]^

#### 3.2.4. Perform AD8 screening.

The AD8 instrument has been validated for assessing dementia prevention but does not examine neurological problems associated with dementia.^[53]^ The AD8 screening is a baseline assessment for older adults to prevent the development of dementia. Moreover, it is a brief and sensitive screening instrument that is associated with earlier and more accurate diagnosis of Alzheimer’s Disease.^[53]^ The AD8 is a valid, reliable, practical, and appropriate scale to distinguish between normal cognitive aging, mild cognitive impairment, and dementia development among older adults.^[54–57]^

### 3.3. Spiritual health promotion with dementia prevention

#### 3.3.1. Have religious beliefs.

Some studies have explored the association between religious beliefs and cognitive decline in dementia prevention. The 3 religious faiths of Taoism, Buddhism, and Christianity have been assessed as effective factors in dementia prevention.^[58]^ An evidence-based review found a significant positive relationship between religious beliefs, spiritual involvement, and fewer mental problems.^[59]^ Religious involvement is correlated with better mental health promotion to cope with mental problems such as depression, stress, anxiety, and dementia.^[59]^ The practice of religious beliefs, religious coping strategies, and/or spirituality decreases depression, thus reducing the development of dementia. Many religious beliefs and spiritual well-being interventions have been demonstrated to have a direct effect on dementia prevention through decreased depression and increased spiritual health.^[60]^ Spiritual health fitness and lifestyle are important contributors to brain longevity and an improved aging process for dementia prevention.^[61]^

#### 3.3.2. Listen to spiritual music.

Music may enhance memory and emotional functions in older adults and contribute to dementia prevention. A previous study reported positive effects of music exposure on cognitive function in dementia prevention.^[62]^ Older adults and their family caregivers should implement music listening interventions to improve mood, stress, anxiety, depression, agitation,^[63]^ and spiritual and social health towards dementia prevention.^[64]^ Listening to spiritual music can increase memory and promote a positive emotional state, which are both associated with dementia prevention among older adults.^[62–64]^

#### 3.3.3. Attend to meditative activities.

The literature shows that practicing effective meditative techniques improves memory, slows cognitive decline, and reduces mild cognitive impairment among older adults.^[65,66]^ Meditative techniques have also been demonstrated to improve numerous other problems, such as sleep disorders, stress, anxiety, and depression, and improve spiritual fitness, spiritual well-being, mental health, cognitive function, and prevention of dementia.^[65,66]^ Meditation is relatively easy to learn and practice in many programs and interventions among older adults and includes mental stimulation, emotional regulation, socialization, attentional regulation, and dementia prevention.^[65,66]^ Meditative intervention has also been shown to be affective in improving cognitive function in older adults in their daily lives.^[67]^ Therefore, meditative and mind-body activities enhance cognitive function and dementia prevention through neural, biological, and behavioral health strategies in older adults.^[67,68]^

#### 3.3.4. Practice mindfulness.

Mindfulness interventions involve meditative techniques and promote cognitive functions associated with neurodegenerative problems in older adults.^[67]^ A mindfulness yoga program in older adults involves stretching and resistance training, reduces stress, anxiety, and psychological distress, and improves physical health, spiritual well-being, and quality of life.^[69]^ Mindfulness incorporated into stress reduction programs is an effective positive coping strategy that enhances spiritual health, social support, and progressive muscle relaxation to decrease anxiety and depression, and improve quality of life.^[69]^ One study assessed the effectiveness of mindfulness among older adults and reported improvements in cognitive function, anxiety, depression, dementia, and mild cognitive impairment to improve quality of life.^[70]^

#### 3.3.5. Do yoga, Qi-gong, Tai-chi, and Baduanjin.

Yoga is one of the most widely used body and mind activities for spiritual health promotion, dementia prevention, and treatment of neurological problems. Yoga may also be considered an effective strategy among older adults to improve psychological distress, cerebrovascular problems, neurological disorders, and spiritual well-being for quality of life.^[71–73]^ A study demonstrated that active walking and practicing Qi-gong in the forest had beneficial effects on neuropsychological health and dementia prevention.^[74]^ In fact, Qi-gong and Baduanjin may be highly beneficial in promoting cognitive ability in older adults for dementia prevention. Baduanjin health activity improves neuropsychological outcomes in terms of dementia prevention.^[75]^ In addition, Tai-chi and Baduanjin activities may enhance memory function among older adults and are correlated with improved mental health promotion, spiritual health promotion, and comprehensive memory to improve memory and preventing dementia.^[76]^ Tai-chi interventions have also been demonstrated to prevent dementia among older adults with improved quality of life.^[77,78]^ Overall, older adults who perform yoga, Qi-gong, Tai-chi, and/or Baduanjin achieve great benefits in terms of dementia prevention and spiritual health promotion.

### 3.4. Social health promotion with dementia prevention

#### 3.4.1. Have a supportive family system.

Social health promotion is a highly effective intervention for preventing dementia in older adults.^[3]^ Such promotion will greatly reduce the global burden of dementia prevention on older adults and their families.^[3,46]^ Older adults living with family members may experience enhanced social health, social support, and a reduced incidence of dementia with improved quality of life.^[46]^ Dementia prevention using the AD8 screening test is also an important approach for a supportive family system among older adults.^[79]^

#### 3.4.2. Enhance socialization.

Social isolation greatly impacts older adults’ life satisfaction and well-being in terms of physical, mental, spiritual, and social health. Older adults may increase their socialization activities, social connections, and social environments to promote dementia prevention and achieve optimal social health.^[80]^ One study demonstrated that poor social engagement, such as poor social networks and poor social support, is associated with an increased probability of dementia among older adults.^[80]^ Effective interventions for social engagement encourage older adults through targeted socialization for dementia prevention and social health promotion.^[80]^

#### 3.4.3. Establish social support.

Social robots are presently a form of assistive technology used to maintain independence, promote well-being, and improve cognitive impairment in older adults in daily life. Social robots may provide social support for older adults to improve the aging process in terms of dementia prevention. Many studies have shown correlations between robots and dementia, between robots and cognitive impairment, between robots and social support, and between robots and the aging process. The proper use of social robots can establish social support in a positive manner and achieve dementia prevention and stress reduction among older adults.^[81]^

#### 3.4.4. Create social networks.

Healthy lifestyles in terms of social networks and social health promotion among older adults may decrease the risk of dementia incidence and increase dementia prevention and management.^[82]^ Social networks with families and friends may greatly influence dementia prevention and improve cognitive function, depression, and mental health among older adults.^[82]^ Older adults’ social networks are a growing topic in the fields of dementia prevention and social health promotion.^[12,83]^

#### 3.4.5. Increase social interactions.

One study showed that frequent social interactions and activities are effective health promotion strategies for preventing dementia in older adults.^[84]^ Social contact and interactions with friends and family members are associated with a lower risk of dementia and constitute a protective factor for cognitive function among older adults. In the future, frequent social interactions will be increasingly used as an effective method to improve cognitive health outcomes, prevent dementia, and promote social health.^[85]^

#### 3.4.6. Promote social participation.

Social participation may reduce the occurrence of dementia by 40% in Taiwan, including community activities, charitable activities, volunteering services, classmate meetings, and religious activities.^[1]^ Since social isolation is a major risk factor for dementia, we need to increase the protective factors against dementia by promoting social participation among older adults. Healthy lifestyles involving social participation must be implemented to reduce social isolation and prevent dementia. Indeed, social participation among older adults has been shown to reduce dementia incidence.^[86]^ Enhancing social participation to improve well-being contributes to preventing dementia in older adults.^[87]^

## 4. Conclusion

The conclusion of this narrative review was to integrate the concepts of dementia prevention and health promotion in the quality of life on 4 aspects associated with physical, mental, spiritual, and social health among older adults. We integrated an overall framework of dementia prevention regarding physical, mental, spiritual, and social health promotion in daily life as follows: physical health promotion, which includes cognitive activities, physical activities, BMI control, a balanced diet, the Rainbow diet, the Mediterranean diet, the DASH diet, the Mind diet, no smoking, no drinking, none of the three highs (i.e., hyperglycemia, hyperlipidemia, and hypertension), and no head injury; mental health promotion, which includes positive thinking, BSRS-5, depression scale, and AD8 screening; spiritual health promotion, which includes religious beliefs, spiritual music, meditative activities, mindfulness, yoga, Qi-gong, Tai-chi, and Baduanjin; and social health promotion, which includes a supportive family system, socialization, social support, social networks, social interaction, and social participation. Overall, we found that interventions that encourage the maintenance of an active brain increased protective factors and decreased risk factors among older adults to promote dementia prevention.

## Author contributions

**Conception:** Fu-Ju Tsai, Sheng-Wei Shen.

**Supervision:** Sheng-Wei Shen.

**Visualization:** Sheng-Wei Shen.

**Writing—original draft:** Fu-Ju Tsai.

**Writing—review and editing:** Fu-Ju Tsai, Sheng-Wei Shen.
